# Differences in the Prevalence of Obesity among Fars-Native, Turkman, and Sisstanish Ethnic Groups in Iranian Northern Adults in 2010

**Published:** 2013-06-01

**Authors:** Gholamreza Veghari, Mehdi Sedaghat, Siavash Maghsodlo, Samieh Banihashem, Pooneh Moharloei, Abdolhamid Angizeh, Ebrahim Tazik, Abbas Moghaddami

**Affiliations:** 1 Department of Nutrition and Biochemistry, School of Medicine, Golestan University of Medical Sciences, Gorgan, IR Iran; 2 Deputy of Health, Golestan University of Medical Sciences, Gorgan, IR Iran

**Keywords:** Obesity, Adults, Ethnic Group, IR Iran

## Abstract

**Objectives:**

The aim of this study was to evaluate the differences of obesity rate among three ethnic groups in northern adults in IR Iran in 2010.

**Methods:**

The present cross-sectional, analytical study was conducted on 2994 cases of the same age and sex in three ethnic proportions (Fars-native=1625, Turkman=977, and Sisstani=392). The subjects aged between 15 and 65 years old and were selected by multistage cluster sampling techniques including 150 clusters each containing 20 subjects in urban and rural areas in 11 districts in Golestan province (northern IR Iran). Obesity was defined after WHO classification by BMI (Body Mass Index) equal or over 30 kg/m^2^. SPSS 16.0 software was used for statistical analysis and *P* value<0.05 was considered as statistically significant.

**Results:**

Mean±SD of BMI in Fars-native, Turkman, and Sisstanish ethnic groups was 26.72±5.56, 26.18±5.34, and 24.59±6.72 kg/m^2^, respectively. Averagely, obesity was common in 22.8% of the subjects and was significantly higher among the females compared to males (32.3% vs13.3%) (*P*=0.001). Also, its prevalence was estimated as 25%, 22.6%, and 14% in Fars-native, Turkman, and Sisstanish ethnic groups, respectively. Statistical differences were significant among the three ethnic groups (*P*=0.001). The risk of obesity was 2.041 [95% CI, 1.502-2.722] in Fars-native and 1.781 [95% CI, 1.298-2.472] in Turkman groups compared to Sisstanish ethnic group.

**Conclusions:**

Over one out of five adults in northern IR Iran suffer from obesity and an alarming rate was shown among the women. Among the three ethnic groups, the highest and the lowest rates were seen in Fars-native and Sisstanish ethnic groups, respectively.

## 1. Background

Over the past decade, obesity has been emerged as a major public health problem due to its role in cardiovascular disease, stroke, and type 2 diabetes (1). According to the World Health Organization report, obesity is increasing worldwide (2). Obesity is well known as a health problem in IR Iran (3), particularly northern IR Iran (4,5). In IR Iran, the prevalence of overweight and obesity in 2005 was 42.8% in males and 57% in females (6). Also, these figures are predicted to be 54% and 74%, respectively in 2015 (7).

Ethnic differences in obesity were detected between African-Americans and whites or Hispanics in the US (8). In western Sydney, the prevalence of obesity was significantly higher among the children from a Pacific Island, Middle Eastern, or European background compared to English speaking background children (9). In Australian population, at the same Body Mass Index (BMI) value, Japanese men had greater body fat deposition in comparison to Australian-Caucasians and lower BMI cut-off points were recommended to identify obese Japanese men (10). Moreover, body dimension and adiposity indices were significantly different among three ethnic groups in India (11).

The body composition of individuals varies among different ethnic groups. Moreover, the socio- economic background on body composition as a constitutional difference in percentage fat mass, has been reported in American and European Caucasians at the same BMI level (12). Some studies have shown differences in the prevalenc of obesity among ethnic groups (4,13,14). Culture and lifestyle can also affect weight management (15,16). Yet, the strong relationship between the socio-demographic factors and obesity was shown in some regions (17,18).

Among the 1.6 million people living in Golestan province (northern IR Iran and south east of Caspian Sea), 66.39% are between 15 and 65 years old and 43.9% live in urban areas. Many ethnic groups, including Fars-native, Turkman, and Sisstani, live in this area and the main job is agriculture in villages (19).

Since no studies have been conducted on the differences of obesity among the three ethnic groups in northern IR Iran, this study was designed and established in this area. The main aim of this study was to evaluate the differences of obesity rate among the three ethnic groups in Iranian northern adults in 2010.

## 2. Methods

### 2.1. Study Design

In a Comprehensive non-Communicable Disease Study in 2010, we established a cross-sectional and analytical study with a sample of 2994 cases of the same age and sex and in three ethnic proportions (Fars-native=1625, Turkaman=977, and Sisstani=392) of urban and rural populations. The subjects aged between 15 and 65 years old and lived in 11 districts in Golestan province, northern IR Iran. Considering the obesity rate of 50% (6), confidence level of 95%, and maximum marginal error of about 0.02, a 2401–subject sample size was determined for the study. Of course, the sample size was increased up to 2994 subjects for more efficiency. The samples were selected using multistage cluster sampling techniques by 150 clusters each containing 20 subjects. In the first stage, the clusters were randomly selected through systematic sampling technique based on the postal code in urban areas and family health number in Primary Health Centers in rural areas. In the second stage, 20 subjects were randomly selected in each cluster. The data were collected during three mounts and six individual were missed after sampling.

### 2.2. Subjects

The present study was conducted on northern individuals in Golestan province (northern IR Iran) who were from 15 to 65 years old and lived in urban and rural areas. Pregnant women and the individuals who were unwilling to participate in this study were excluded from the study.

A trained staff recorded the data using a multidimensional questionnaire including socio- demographic indexes during three mounts.

### 2.3. Anthropometric Measurement Technique

Body-weight was measured to the nearest 0.1 kg. Besides, height was measured to the nearest 0.5 cm with the subjects standing up and the head, back, and buttocks on the vertical area of the height-gauge.

### 2.4. Variables Definition

BMI was calculated as weight in kilograms (kg) divided by height in meters squared. BMI of 25.0-29.9 kg/m2, 30.0 to 39.9 kg/m2, and equal to or greater than 40 kg/m2 were classified as overweight, obese, and pathologically obese, respectively (20).

The ethnic groups in this study were divided to three groups: 1) Fars-native: This ethnic group includes the natural inhabitants of this province, 2) Turkman: Intermarriage between this ethnic group and other ethnic groups was rare; therefore, this ethnic group can be considered as a pure race, and 3) Sisstani: These individuals immigrated from Sistan and Baluchistan province (east of IR Iran) many years ago.

### 2.5. Statistical Analysis

Quantitative and qualitative data are presented as mean ± standard deviation and frequency percentage respectively. All the statistical analyses were performed using the SPSS statistical software (v. 16). ANOVA and post-hoc Tukey test were used to compare the means and the frequencies were compared through Chi-square test. In addition, logistic regression analysis was applied to estimate the Odds Ratio (OR) of obesity in the ethnic groups according to gender. Besides, *P* value<0.05 was considered as statistically significant. This study was approved by the Ethical Research Committee and written informed consents were obtained from all the participants.

## 3. Results

The mean age of the study subjects was 39.2±11.6 years and the distribution of ethnicity was

54.3% in Fars-native, 32.6% in Turkman, and 13.1% in Sisstanish ethnic groups. Moreover, the mean of BMI was 26.72, 26.18, and 24.59 kg/m2 in Fars-native, Turkman, and Sisstanish ethnic groups, respectively and the difference was statistically significant (*P*=0.001).

The results of ANOVA revealed a significant difference among both males (*P*=0.001) and females (*P*=0.029) of the 3 ethnic groups regarding the mean of BMI. In males, the post-hoc test was significant between Fars-native and Turkman groups (*P*=0.006) as well as Turkman and Sisstani groups (*P*=0.001); however, it was not significant in women. Considering the females, the results of ANOVA revealed a significant difference between Fars-native and Sisstani (*P*=0.010) (Table 1).

**Table 1.** Mean and SD of Age and BMI among the Three Ethnic Groups Based on Gender

Ethnicity	Gender	Age (year)	BMI(Kg/m^2^)	*P* value[Table-fn fn5163]
		mean(SD)	mean(SD)	
Fars-native	Men(814)	39.86(14.42)	25.65(4.91)	0.001
	Women(811)	40.01(14.06)	27.80(5.94)	
	Total(1625)	39.93(14.23)	26.72(5.56)	
Turkman	Men(488)	40.15(14.22)	24.89(4.46)	0.001
	Women(489)	39.63(14.22)	27.47(5.82)	
	Total(977)	39.89(14.22)	26.18(5.34)	
Sisstani	Men(197)	39.37(14.75)	22.71(4.63)	0.001
	Women(195)	39.42(14.07)	26.49(7.88)	
	Total(392)	39.40(14.39)	24.59(6.72)	
Total	Men(1499)	39.89(14.38)	25.02(4.82)	0.001
	Women(1495)	39.81(14.12)	27.52(6.20)	
	Total(2994)	39.85(14.24)	26.27(5.69)	

Post-hoc.test between Fars-native and Turkman in men (P=0.006) and in whole (P=0.015) is significant but in women is not significant (P=0.320). Post-hoc. test between Fars-native and Sisstani in men (P=0.001), women (P=0.010) and in whole (P=0.001) is significant. Post-hoc. test between Turkman and Sisstani in men (P=0.001) and in whole (P=0.001) is significant but in women is not significant (P=0.075). ANOVA among three ethnic groups in men (P=0.001), in women (P=0.029) and in whole (P=0.001) is significant. There is no statistical signification in internal ethnic groups and among ethnic groups based on age.

Overall, obesity was detected in 22.8% of the subjects and its prevalence was estimated as 25%, 22.6%, and 14% in Fars- native, Turkman, and Sisstanish ethnic groups, respectively (*P*=0.001). Moreover, the prevalence of obesity among the females was 19% higher than males (*P*=0.001) and it was significant between genders in three ethnic groups (*P*=0.001). Nevertheless, no significant differences were found between Fars-native and

Turkman ethnic groups based on males, females, and on the whole (Table 2).

**Table 2.** The Comparison of Obesity among the Three Ethnic Groups Based on Gender

Ethnicity				BMI Distribution					Total[Table-fn fn5164]
				≤18.5	18.5-25	25-30	30-40	40≤	
Fars- native	Gender		Men(814)	30(3.7)	370(45.5)	289(35.5)	116(14.3)	9(1.1)	0.001
			Women(811)	30(3.7)	244(30.1)	256(31.6)	259(31.9)	22(2.7)	
	Total(1625)				60(3.7)	614(37.8)	545(33.5)	375(23.1)	31(1.9)	
Turkman	Gender		Men(488)	27(5.5)	233(47.7)	166(34.0)	59(12.1)	3(0.6)	0.001
			Women(489)	25(5.1)	163(33.3)	142(29.0)	149(30.5)	10(2.0)	
	Total(977)				52(5.3)	396(40.5)	308(31.5)	208(21.3)	13(1.3)	
Sisstani	Gender		Men(197)	30(15.2)	116(58.9)	39(19.8)	11(5.6)	1(0.5)	0.001
			Women(195)	13(6.7)	83(42.6)	56(28.7)	37(19.0)	6(3.1)	
	Total(392)				43(11.0)	199(50.8)	95(24.2)	48(12.2)	7(1.8)	
Whole	Gender		Men(1499)	87(5.8)	719(48.0)	494(33.0)	186(12.4)	13(0.9)	0.001
			Women(1495)	68(4.5)	490(32.8)	454(30.4)	445(29.8)	38(2.5)	
Total (2994)				155(5.2)	1209(40.4)	948(31.7)	631(21.1)	51(1.7)	

Chi-2 between Fars-native and Turkman in men (P=0.218), women (P=0.431) and in whole (P=0.172) is not significant. Chi-2 between Fars-native and Sisstani in men (P=0.001), in women (P=0.001) and in whole (P=0.001) is significant . Chi-2 between Turkman and Sisstani in men (P=0. 012), in women and in whole (P=0.001) is significant. Chi-2 among three ethnic groups in men (P=0.002), in women (P=0.003) and in whole (P=0.001) is significant. Chi-2 between genders in three ethnic groups and in whole is significant (P=0.001 for all).

The results of logistic regression analysis showed that the risk of obesity was 2.041 [1.502-2.722] in Fars-native and 1.781 [1.298-2.472] in Turkman group compared to Sisstanish ethnic group. In addition, the risk of obesity was respectively 2.797 [1.513-5.169] and 2.244 [1.181-4.263] in Fars-native and Turkman males compared to Sisstanish ethnic group. Considering the females, the risk of obesity in Fars-native and Turkman groups was respectively 1.847 [1.297-2.708] and 1.703 [1.156-2.510] compared to Sisstanish ethnic group (95% CI for all) (Table 3).

**Table 3.** The Results of Logistic Regression Analysis Estimated the Odds Ratio of Obesity in North of IR IR Iran (95% CI)

Variable		Odds Ratio (Lower Limit­ Upper Limit)	*P* value
Total	Sisstani	Ref(1)	­
	Fars­native	2.041(1.502­2.722)	0.001
	Turkman	1.791(1.298­2.472)	0.001
Men	Sisstani	Ref(1)	­
	Fars­native	2.797(1.513­5.169)	0.001
	Turkman	2.244(1.181­4.263)	0.014
Women	Sisstani	Ref(1)	­
	Fars­native	1.874(1.297­2.708)	0.003
	Turkman	1.703(1.156­2.510)	0.007

## 4. Discussion

In present study, obesity was detected in one out of five adults in northern IR Iran and it was more prevalent among the females compared to males. Obesity was different among the three major ethnic groups in this area.

Obesity and overweight were prevalent in 18.1% and 32.0% of adults in IR Iran, respectively (6). In another study (21), the prevalence of overweight, obesity, and pathological obesity was reported as 28.6%, 10.8%, and 3.4% of adults, respectively.

In Semnan (a province in center of IR Iran), overweight and obesity were seen in 40.6% and 26.3% of adults, respectively (22). In a systematic-review and meta-analysis also, obesity was reported in 13.7% of males and 27.3% of females (23).

Obesity is substantially unequal in the world and its current prevalence ranges from as low as ≤ 5% in China, Japan, and Africa to as high as ≥75% in urban Samoa (24). The prevalence of obesity was reported as 22.9% in Spain (25) and 40% in South Asians countries (26).

Similar to the results of the current study, in almost all the countries, women are more likely to be obese compared to men (27, 28). In the same calorie intake, men tend to gain less weight than women because of more Lean Body Mass and more physic activities that lead to burning more calories in men.

In present study, the highest and lowest odd ratios for obesity were related to Fars-native and Sisstanish ethnic groups, respectively.

In general, nutrition behavior is influenced by some biological, cultural, and socio-economic factors (29). The association between nutrition and ethnicity or different immigrant groups in a community has been reported worldwide. In the United States (30), non-Hispanic blacks had the greatest rates of obesity (35.7%) compared to Hispanics (28.7%) and non-Hispanic whites (23.7%) (13). Besides, the prevalence of obesity was higher in Latino people (38.7%) in comparison to non-Latino whites (32.8%) (31). Furthermore, the role of genetic factors on the secular growth was approved in Sri Lanka Australian children (32). Rush (33) recommended using Free Fat Mass (FFM) instead of BMI in a filed study. Moreover, Fredriks (34)recommended separated growth chart for Moroccan and Turkish children whose living in Netherland. In addition to ethnicity, obesity is different among the migrant populations worldwide. For instance, a negative impact on family unity and meal structure was reported among the Mexican immigrant males (14). Also, less access to physical activity facilities and more expensive healthy foods were observed in minority and low-income populations (35). Overall, environmental (high density of fast food establishments) (36) and behavioral (Low rates of physical activity, high intake of energy-dense foods) (37) factors were the main causes of obesity in Southeastern US.

Marked differences in body dimension, adiposity indices, and cardio-respiratory health were seen among three ethnic groups in Indian population (11). Lifestyle also plays a critical role in the obesity rate. In western Sydney, ethnicity and socio-economic status are independently associated with the prevalence of unhealthy weight in the children (9). In general, walking and bicycling are far more common in European countries compared to the United States, Australia, and Canada (38). Furthermore, African-Americans were significantly less likely than whites or Hispanics to view obesity as a health problem (8).

Veghari (39) stated that Turkman children had a better nutritional status compared to Sisstanish ethnic group in northern IR Iran and the prevalence of obesity in Sisstanish women was the lowest in comparison to the ethnic groups in this area (4). Sisstanish ethnic group has immigrated from the east to the north of IR Iran during the last decades and their socio-economic indexes are quite low. Men mainly do physical work, while women are mostly housewives. Of course, more studies are necessary to be conducted on the reasons for the low prevalence of obesity in Sisstanish ethnic groups.

According to the results of the present study regarding the comparison between males and females, the obesity rate was different among the three ethnic groups. Food behavior differences between genders have also been reported in other studies. In Alabama, the role of culture and tradition in weight management was emphasized. Yet, low access to fresh fruits and vegetables and low physical activities were mentioned to play a role in Latino immigrant women (15). In the US, Latino women were more than others affected by obesity (31) and non-Hispanic black women were more satisfied with their body size compared to non- Hispanic white women. The individuals who are satisfied with their body size are less likely to try to lose weight (40). In line with the above-mentioned studies, the obesity differences between genders within ethnic groups was not similar in the current study.

Within the ethnic groups, the risk of obesity was higher in males compared to females and why the gender difference in Sisstanish ethnic group is greater than the others is not clear. Probably, environmental and behavioral factors are various in each of the ethnic groups and this should be considered in future studies.

One of the limitations of the present study was that we did not evaluate many socio-demographic factors affecting obesity, including physical activity, genetics, food behavior, diet, and body fat percent.

## 5. Conclusion

IR Iran is considered to be in the nutrition transition phase and life style and food behavior have changed in the recent years (41). Similar to other developing countries, obesity is a health problem in IR Iran. The findings of the present study showed that over one out of five adults in northern IR Iran suffered from obesity and an alarming rate was observed in females. Among the three ethnic groups, the highest and the lowest rates were related to Fars-native and Sisstanish ethnic groups, respectively. Within the ethnic groups, the risk of obesity in males was higher than women.
